# Implementing Ac-225 labelled radiopharmaceuticals: practical considerations and (pre-)clinical perspectives

**DOI:** 10.1186/s41181-024-00239-1

**Published:** 2024-02-06

**Authors:** Eline L. Hooijman, Valery Radchenko, Sui Wai Ling, Mark Konijnenberg, Tessa Brabander, Stijn L. W. Koolen, Erik de Blois

**Affiliations:** 1 Department of Radiology and Nuclear Medicine, Erasmus MC, 3015 CN Rotterdam, The Netherlands; 2 Department of Hospital Pharmacy, Erasmus MC, 3015 CN Rotterdam, The Netherlands; 3 Life Sciences Division, TRIUMF, Vancouver, BC V6T 2A3 Canada; 4 Chemistry Department, University of British Columbia, Vancouver, BC V6T 1Z1 Canada; 5 Department of Medical Oncology, Erasmus MC Cancer Institute, 3015 CN Rotterdam, The Netherlands

**Keywords:** Targeted alpha therapy (TAT), Targeted radionuclide therapy (TRT), Radiolabelling, Actinium-225, Quality control, Practical implementation, Radiopharmaceutical, GMP production

## Abstract

**Background:**

In the past years, there has been a notable increase in interest regarding targeted alpha therapy using Ac-225, driven by the observed promising clinical anti-tumor effects. As the production and technology has advanced, the availability of Ac-225 is expected to increase in the near future, making the treatment available to patients worldwide.

**Main body:**

Ac-225 can be labelled to different biological vectors, whereby the success of developing a radiopharmaceutical depends heavily on the labelling conditions, purity of the radionuclide source, chelator, and type of quenchers used to avoid radiolysis. Multiple (methodological) challenges need to be overcome when working with Ac-225; as alpha-emission detection is time consuming and highly geometry dependent, a gamma co-emission is used, but has to be in equilibrium with the mother-nuclide. Because of the high impact of alpha emitters in vivo it is highly recommended to cross-calibrate the Ac-225 measurements for used quality control (QC) techniques (radio-TLC, HPLC, HP-Ge detector, and gamma counter). More strict health physics regulations apply, as Ac-225 has a high toxicity, thereby limiting practical handling and quantities used for QC analysis.

**Conclusion:**

This overview focuses specifically on the practical and methodological challenges when working with Ac-225 labelled radiopharmaceuticals, and underlines the required infrastructure and (detection) methods for the (pre-)clinical application.

## Background

In recent years, the interest in targeted radionuclide therapy (TRT) has grown remarkably. TRT targets tumor-specifically, and is thereby very much suited for treating metastasized cancer, showing encouraging results in several types of cancer (Becx et al. 2022). The development of TRT for a specific target is dependent on the biological vector (e.g. small molecules, peptides, and monoclonal antibodies), as well as their conjugation to a specific type of radionuclide (α, β- and Auger). Thereby, the characteristics of the radionuclide and the biological vector could influence the treatment effectiveness, based on the biological effect and biodistribution which is related to the pharmacokinetics and the biological half-life (Nelson et al. 2020). The choice of a radionuclide for (pre-)clinical applications demands careful consideration of its (bio-)chemical and physical characteristics, including factors like the (biological) half-life, daughter nuclides, production methods, and stability. These aforementioned characteristics can lead to a significant variation in therapeutic outcome.

Although the radionuclide therapy using beta-emitters is particularly suited to deliver high radiation dose to the tumor tissue, the therapeutic effect is in some cases limited even after several treatment cycles (Ling et al. 2022). As described in the literature (Seo 2019; Kratochwil et al. 2019; Ruigrok 2022; Scheinberg and McDevitt 2011; Graf et al. 2014), alpha particle emission provides an advantage over beta-emission due to the high linear energy transfer (LET) and the short tissue penetration (50–100 µm) which results in localized dose effects and high local toxicity (Nelson et al. 2020). Initial studies tentatively show that patients who have developed radio-resistance to for example [^177^Lu]Lu-PSMA-617, can still benefit from treatment with targeted alpha therapy (TAT) using [^225^Ac]Ac-PSMA-617 (Ling et al. 2022; Feuerecker et al. 2021; Okamoto et al. 2021). Currently, different alpha-emitters are implemented in a clinical setting, such as [^223^Ra]RaCl_2_ (Xofigo®), [^212^Pb]Pb-DOTAMTATE and [225Ac]Ac-PSMA/ [225Ac]Ac-DOTA-TATE (Kratochwil et al. 2018, 2019; Feuerecker et al. 2021; Filippi et al. 2020; Hooijman, et al. 2021; Lunger et al. 2021; Ma et al. 2022; Morgenstern et al. 2018; Shi et al. 2022), in this manuscript, we focus on Ac-225 specifically.

**Fig. 1:**
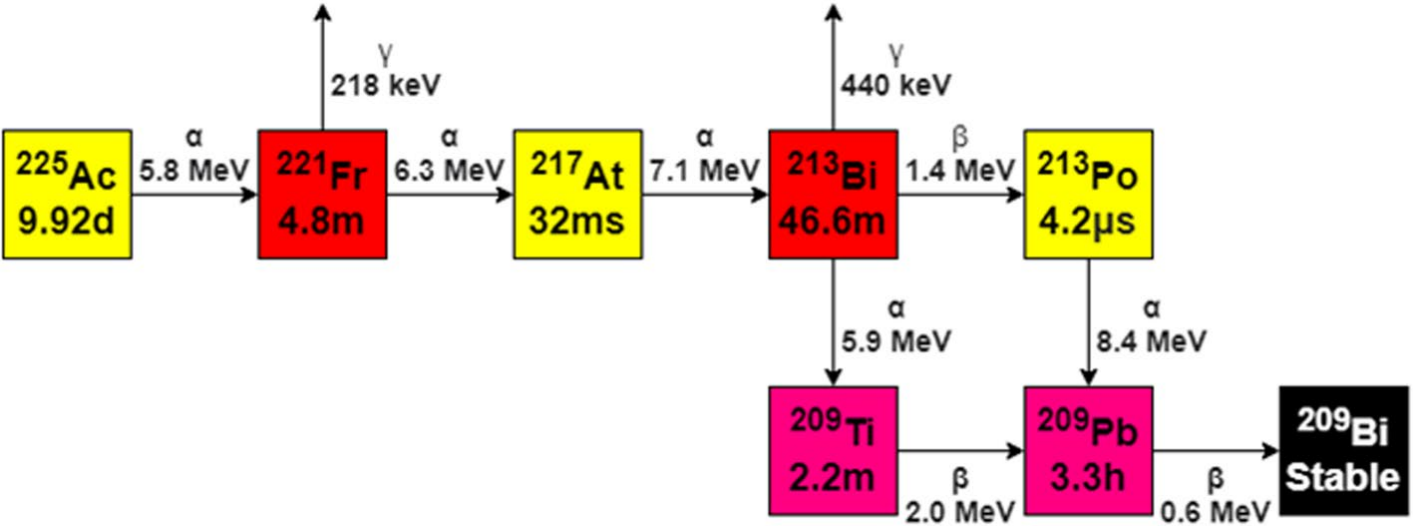
Decay scheme of Ac-225 to near-stable Bi-209

Research interest into alpha emitters is increasingly focusing on Ac-225. It has a physical half-life of 9.92 days, and decays via a cascade of six short-lived radionuclide daughters to Bismuth-209 (Bi-209) that is considered stable (half-life of 2.01 × 10^19^ years). Through decay, Ac-225 emits five alpha particles per decay (Fig. 1), with energies ranging from 5.8 to 8.4 mega-electron volt (MeV) having a tissue range of 47 to 85 µm (µm). The high LET effectively kills tumor cells through double strand DNA breaks and cluster breaks. Additionally, the limited range of the alpha particles increases the energy release to tumor cells while sparing healthy tissue (Ruigrok 2022; Graf et al. 2014). The decay cascade also includes beta disintegrations ranging from 0.6 to 2.0 MeV and gamma co-emissions from Francium-221 (Fr-221) and Bismuth-213 (Bi-213), which are useful for imaging and quality control, see Table 1 (Kratochwil et al. 2019, 2021; Hooijman et al. 2021; Lunger et al. 2021; Morgenstern et al. 2018; Santos et al. 2019; Working et al. 2018; Królicki et al. 2020; Makvandi et al. 2018; Allen 2013, 2008).

**Table 1:** Decay of Ac-225 to daughter nuclides and their emission/yield

Decaying isotope	Half-life (t1/2)	α emission*	β emission	γ emission
Ac-225	9.92 d	5.8 MeV	x	150 keV (0.6%) 188 keV (0.45%)
Fr-221	4.8 m	6.3 MeV	x	218 keV (11.4%)
At-217	32 ms	7.1 MeV	x	x
Bi-213	46.6 m	5.9 MeV (2.1%)	1.4 MeV (97.8%)	440 keV (25.9%)
Po-213	4.2 µs	8.4 MeV	x	x
Tl-209	2.2 m	x	2.0 MeV	x
Pb-209	3.3 h	x	0.6 MeV	x
Bi-209	Stable	x	x	x

*α-Emission leads to recoil from the radiopharmaceutical construct, all daughter nuclides from Ac-225 are ejected outside of the chelate upon Ac-225 decay, see “Equilibrium” section

There are specific aspects that need to be considered when working with Ac-225 labelled radiopharmaceuticals in a clinical setting. Within Europe, the same guidelines and regulations apply for both registered and non-registered radiopharmaceuticals (Lange et al. 2015), as described in the European Pharmacopoeia, Good Radio-Pharmacy Practice (GRPP), guidelines described by the European Association of Nuclear Medicine (EANM) (Gillings et al. 2020, 2021) and EU GMP regulations described by the European Commission. Additionally, health physics regulations apply and as Ac-225 has a high toxicity, limitations for handling and quality control (QC) introduces challenges. In consensus, these guidelines describe that for quality control at least 1% of each impurity should be detected, which is a major challenge for a quantitative detection and is described in more details within this manuscript.

Due to the toxicity of Ac-225 radiopharmaceuticals, establishing optimal labelling conditions and quality control (QC) processes is crucial. Therefore, this paper aims to evaluate Ac-225 labelled radiopharmaceuticals, emphasizing the practical challenges. This manuscript encompasses the exploration of radiochemistry and methodology, starting from preclinical radiolabelling and extending to the complexities of clinical implementation, quality control procedures, patient administration, and waste management protocols.

## Main text

### Radionuclide production of Ac-225

The use of Ac-225 for TAT is rising, creating a higher demand for Ac-225. Hence, there is a necessity to expand production and enhance availability, as these currently pose significant constraints on initiating large-scale clinical trials aiming to demonstrate survival benefits (Radchenko et al. 2021).

The main source of Ac-225 over the past several decades is derived from stocks of Th-229 (T1/2 7920 years) extracted from Uranium-233 (U-233) (t^1^/_2_ 160,000 years). Such stocks are available in the Russian Federation, Germany, and the USA. These batches are currently available in several institutions; Institute for Physics and Power Engineering (IPPE), Obninsk, Russian Federation (Rosatom; Kotovskii et al. 2015), Institute for Transuranium Elements (ITU), Karlsruhe, Germany (Apostolidis et al. 2005a), Oak Ridge National Laboratory (ORNL; Boll et al. 2005), TN, USA (US Department of Energy), and within small/research quantities are also available at Canadian Nuclear Laboratories, Chalk River, Canada (Perron et al. 2020) and Belgian Nuclear Research Centre (SCK CEN), Belgium (Boden et al. 2017).

Due to the half-lives of Th-229 and the first decay daughter Ra-225 (t^1^/_2_ 14.9 days), the regular elution schedule of the generators results in approximately 63 GBq (1.7 Ci)/year, which is below the current demand for clinical trials, preclinical research, and other research needs (Robertson et al. 2018a). Therefore, alternative sources of Ac-225 production were actively investigated over the past decade. One possible solution is to increase the source of Th-229 by extracting it from U-233, which is currently ongoing (TerraPower 2023).

Another production route, which could provide additional quantities of Ac-225 and can significantly increase the availability, is the spallation reaction with high-energy protons on Th-232. Theoretically, a single 10-day irradiation can produce comparable quantities of Ac-225 that are annually available from Th-229 decay. The main limitation for this production route is the required proton energy (≥ 100 MeV) which is only available in a few facilities globally and even fewer available facilities that are actively pursuing this production (namely four to date: Los Alamos and Brookhaven National Laboratories (US DOE (Griswold et al. 2016)), TRIUMF, Canada (Robertson et al. 2020) and Institute for Nuclear Research, Russian Academy of Science, Troitsk, Russian Federation (Zhuikov et al. 2011)).

When Ac-225 is produced by spallation, it contains isotopic contamination of Ac-227 (0.1–0.3% activity of Ac-225 at the end of bombardment (EOB) (Weidner et al. 2012)) which can present both clinical limitations and waste disposal problems as in many countries limits for disposal of Ac-227 is very low due to the long half-life (t^1^/_2_ = 21.8y). To overcome this issue TRIUMF pursuing the extraction of radium isotopes parallel with actinium isotopes and the elution of isotopically pure Ac-225 from the decay of Ra-225 (Robertson et al. 2020). Mass separation of Ac-225 is another approach to produce isotopically pure Ac-225 derived from the spallation of Th or U-isotopes and research is ongoing at TRIUMF (Ramogida et al. 2019) and CERN (Johnson et al. 2023).

Two other production strategies represent the significant potential to increase the supply of Ac-225/Bi-213. Both productions are based on irradiation of Ra-226 (t^1^/_2_ 1600 years) with medium energy protons (15–20 MeV) and photons. Theoretically, medium energy proton irradiation can result in comparable yields to the previously discussed thorium-spallation route with much lower co-production of Ac-227 and other actinium-isotopes (Apostolidis et al. 2005b; Nagatsu et al. 2021). However, a significant advantage over the spallation reaction and other production strategies is the wide availability of medium-energy cyclotrons worldwide (IAEA 2023). The photonuclear production route results in a lower production yield, which can be compensated by increasing the target mass, which results in the accumulation of Ra-225 which can be used as a generator for Ac-225 (Grimm et al. 2019). To date, only proof of principles experiments were reported for proton and photon production routes. The main challenges for these production routes are securing enough Ra-226 and developing technology for safe irradiation of highly radioactive targets (especially radon mitigation).

Several other production methods can be considered for the research-level production of Ac-225 which was discussed in detail by Robertson et al. (Robertson et al. 2018a). The radionuclidic purification of Ac-225 from the irradiated target as well as from parent Th-229, is an important aspect for synthesis of radiopharmaceuticals, which was discussed in the review by Ferrier et al. in detail (Maryline et al. 2019).

## Radiochemistry of Ac-225 radiopharmaceuticals

### Radionuclide preparation and source

Radionuclide preparation is crucial, and the quality, including both radionuclidic (“HP-Ge detector” section) and chemical purity of the Ac-225 source, must be assessed before its application in experiments. To date, Ac-225 is only available as a non-GMP product, which makes the testing of impurities even more important.

Ac-225 is commonly supplied as a dry substance, which is preferred as after dilution of the activity, a high flux of radicals is formed by the radiolysis of the present water, which introduces impurities (Blois et al. 2012; Garrison 1987). Furthermore, impurities are often present in the form of metal-ions, such as Iron (Fe), Zinc (Zn), and Copper (Cu), and are dependent on the used target material and production route and materials used for production and shipping. Working metal free is of high importance to maximize the labelling efficiency (Blois et al. 2012, 2014). Tools and columns used in the process could introduce metal impurities.

A stock of Ac-225 can be prepared by dissolving the activity in an acidic solution (i.e. 0.1 M HCl). The stock should be used after > 30 min, to ensure appropriate dissolving. In our experience, after dilution in a V-shaped quartz coated vial (10 mL, Curium, Petten, The Netherlands), the activity can be used for up to two weeks. As the activity will decrease due to decay, and the proportion of stable impurities will remain constant or even increase, the solution will thereby not be suitable for labelling anymore since lower RCY will be obtained. In addition, the ingrowth of impurities is vial-dependent and should be evaluated before using the source. Therefore, for waste management and clinical use, the radionuclide purity has to be determined on beforehand and should be part of the validation phase (“HP-Ge detector” section).

### Chelators

Actinium is the lightest actinide and the largest + 3 cation in the periodic table with an ionic radius of 1.12 Å (Katz 2010). Studies of coordination and complexation preferences of actinium are challenged by the fact that there are no stable isotopes of actinium available with the longest-lived isotope represented by Ac-227 (t1/2 = 21.7 years). Several comprehensive reviews have been recently published discussing latest advancement in the chelation of actinium-225 (Thiele and Wilson 2018; Yang et al. 2022). Notably, actinium does not hydrolyze below pH 9, while most of the 3+ cations will form hydroxides at lower pH values. Therefore, this can be practically applied to performing radiolabelling at higher pH to avoid competing with other cations for the chelation.

**Fig. 2:**
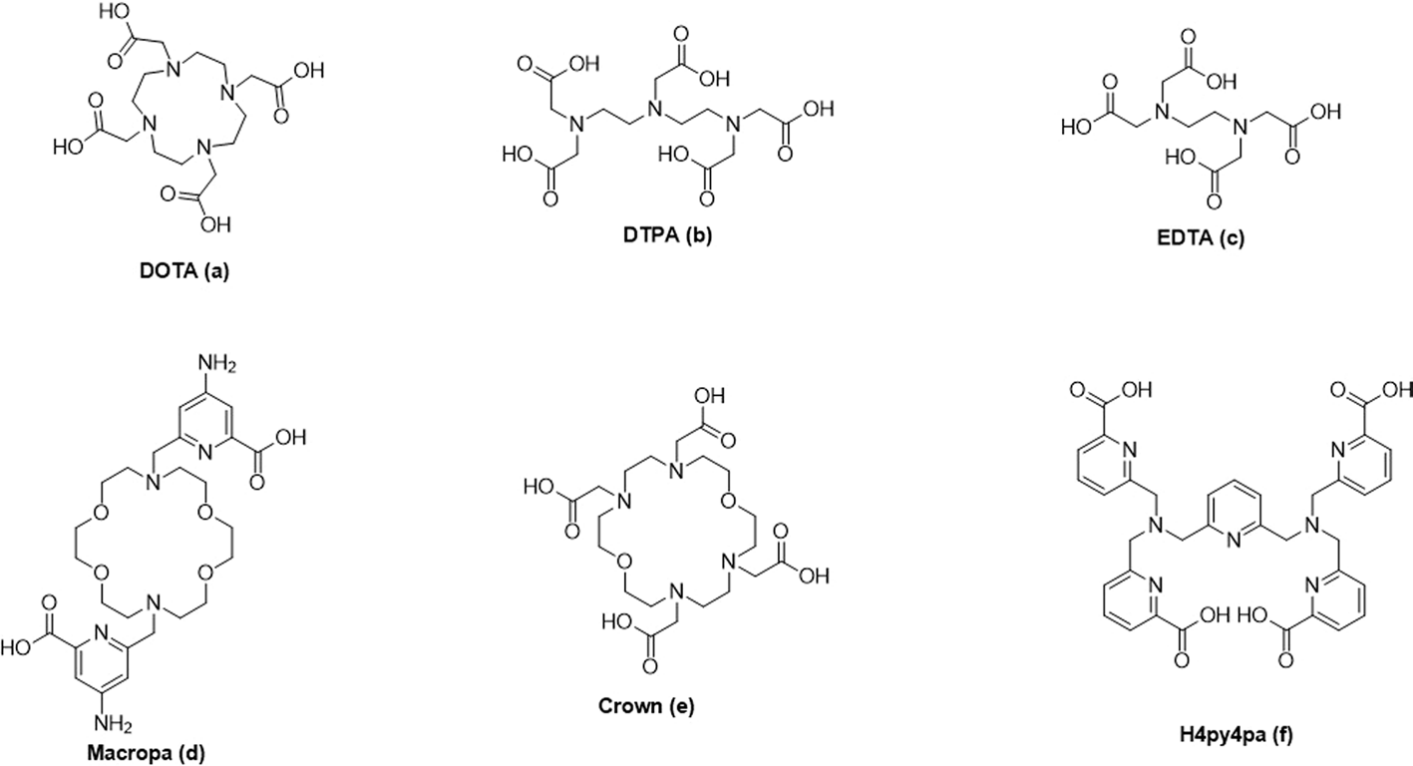
Chelators for Ac-225 complexation; DOTA (**a**), DTPA (**b**), EDTA (**c**), Macropa (**d**), Crown (**e**) and H4py4pa (**f**)

The most commonly used chelators in nuclear medicine are the DOTA ((1,4,7,10-tetraazacyclododecane-1,4,7,10-tetraacetic acid), DTPA (Diethylenetriaminepentaacetic acid) and EDTA (Ethylenediaminetetraacetic acid) chelators, their structure is shown in Fig. 2 (Thiele and Wilson 2018; Rubira et al. 2023; Eychenne et al. 2021). Due to the characteristics of the DOTA chelator, complexation with Ac-225 as well as the wide availability and known toxicity it often the best option for clinical implementation and is therefore widely used (Thiele and Wilson 2018). However, when the DOTA conjugated biological vector is heated, the stability of the structure may be influenced. DTPA and EDTA have proven not to be effective as chelators for Ac-225, due to poor stability. Complexation of Ac-225 and EDTA was studied and showed and increased uptake in the liver and bone (Deal et al. 1999). Therefore, both chelators are currently mostly used for the capture of unlabelled or daughter nuclides after radiolabelling as no additional heating step is required (Breeman et al. 2003, 2005).

Many research groups are currently developing new, or optimized chelators for a stable complexation of Ac-225 (Yang et al. 2022). One of the chelators of interest is the H2macropa (Macropa, Fig. 2), and has shown to form a stable complex with large lanthanide ions. This chelator enables complexation in 5 min at room temperature at low concentrations (Thiele and Wilson 2018), showing high selectivity and ability to retain the Ac-225 (Thiele et al. 2017; Kadassery et al. 2022). However, King et al. described that the advantages in vivo over DOTA are limited (King et al. 2023). In an attempt to increase the yield and reduce the difficulties of the synthesis, other variants of the Macropa chelator were developed, unfortunately, demonstrating a decrease in stability (Kadassery et al. 2022).

Additionally, the recently developed Crown chelator, is capable of the complexation with Ac-225 in 15 min at room temperature with a high molar activity (Yang et al. 2020). Moreover, the H4py4pa chelator (Li et al. 2021) enables complexation also at room temperature, with a high stability (Fig. 2). Consequently, there is a need for in vivo studies and implementation towards the clinic.

Further theoretical computational modelling suggests that the approach should be focused on the identification of the ionic bonding interactions, maximizing the electrostatic interactions (Yang et al. 2020, 2022; Thiele et al. 2017; Li et al. 2021; Morgenstern et al. 2021; Price and Orvig 2014; Kelly et al. 2017).

For example, heat sensitive constructs may benefit from a labelling with a chelator at room temperature (Macropa, Crown, H4py4pa). As Thiele et al. stated, the most prominent challenge when developing a chelator for the retention of Ac-225 3^+^-ion is the recoil effect, whereby due to the high energy of the alpha-emission, the decay product of Ac-225 is released from its chelator (see “Equilibrium” section). Optimizing the stability regarding reducing recoil is limited due to the high energy upon decay. Liposomes and polymersomes may be an alternative to retain the Ac-225 daughters after recoil (Kruijff et al. 2019; Mdanda et al. 2023).

Additionally, the different chemical properties of the daughter nuclides can lead to difference in the in vivo biodistribution. The release of Fr-221 from the chelator after Ac-225 decay, leads to the free circulation of its daughters, which may introduce off-target radiotoxic effects (Thiele and Wilson 2018; Kruijff et al. 2017; Cędrowska et al. 2018).

The efficiency of labelling Ac-225 with the chelators described here may depend on the production and purification method of the Ac-225 source. The Ac-225 that is used for labelling (production from a Th-229 source), can be considered non-carrier added. However, when considering other production method that co-produce Ac-227 (0.1–0.3%, T1/2 21.8y), the contribution of Ac-227 in moles is higher than might be expected. For a sample containing 10 MBq of Ac-225 and 0.3% Ac-227, ratio of both nuclides is a factor 2 (~ 20 vs. ~ 50 pmol Ac-225/Ac-227). In combination with the already present metal-ion impurities, higher negative impact on the radiochemical yield (RCY) is expected.

### Biological vectors

**Fig. 3:**
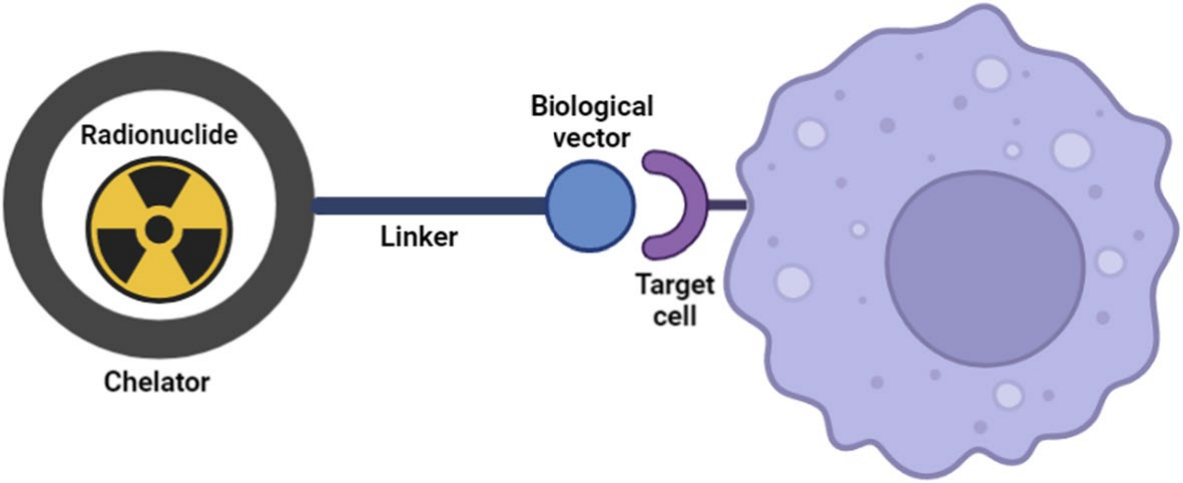
General overview of a radiopharmaceutical construct

In recent years, different Ac-225 labelled biological vectors are described and reviewed in literature (Hatcher-Lamarre et al. 2021; Kim and Brechbiel 2012). Choosing the right biological vector for Ac-225 radiopharmaceuticals is highly dependent on tumor retention and biodistribution, and therefore influences the potential patient safety. The radiopharmaceutical construct consists out of a radionuclide complexed with a chelator, coupled with a linker to the biological vector which binds to the target cell (Fig. 3). Here we focused on radiometal radiochemistry specifically. Although multiple studies reported on the therapeutic effectivity of Ac-225, the detailed description of the related radiochemistry thereof is limited.

The first reported studies using Ac-225 were focused mainly on Ac-225 labelled monoclonal antibodies, thereby showing promising results for leukemia, lymphoma, breast cancer, ovarian cancer, and neuroblastoma (McDevitt et al. 2001; Miederer et al. 2008; Jurcic 2020; Arndt et al. 2022). Additional research on Ac-225 labelled antibodies showed encouraging results for recurrent glioblastoma (Królicki et al. 2020), solid tumors (Cheal et al. 2020), prostate cancer (Arndt et al. 2022), lung tumors (Kennel et al. 2000) and primary liver carcinoma (Bell et al. 2020). When working with an antibody for TAT, it is important to consider the biological, as well as the physical half-life. As antibodies generally have a long circulating time, the absorbed dose for sensitive organs may reach a critical level, thereby increasing the risks of side effects in for example the bone marrow (Hamacher and Sgouros 2001). Furthermore, as the circulation time increases, the incidence of recoil also increases, of which the effect is yet to be investigated further (Carter et al. 2018). However, the high precision of the targeting as well as a specific mechanism of action may minimize other side effects.

Alternative strategies include nanobodies (Urbanska et al. 2020; Salvanou et al. 2020; Ruggiero et al. 2010; McLaughlin et al. 2013, 2014; Bandekar et al. 2014; Matson et al. 2015), antibody fragments, small molecules or peptides. Hereby, rapid elimination can reduce toxicity; nevertheless, striking a balance between the elimination rate and delivering the appropriate dose is essential for optimizing safety and effectiveness in clinical applications. Additionally, a limited radiation dose delivery due to a short biological half-life may provide a challenge.

Recent clinical interest is mainly focused on the treatment of prostate cancer (Thakral et al. 2021; Tagawa et al. 2018), as well as in labelled somatostatin analogs for neuroendocrine tumors (Kratochwil et al. 2021; Kamaleshwaran et al. 2020; Harris and Zhernosekov 2022), thereby showing encouraging therapeutic effects. It is important to note that due to the high toxicity, the biodistribution and related delivered dose should be well evaluated and well considered before applying an alpha radionuclide like Ac-225. Uptake in other organs than the target may lead to serious side effects (Ma et al. 2022). Pretargeting may provide an option to limit the off-target dose while maintaining the specificity, as the biological vector is injected firstly to ensure the specific binding, followed by the radioactive vector for a treatment efficacy (Verhoeven et al. 2019).

Internalization is a process which may play a key role within targeted radionuclide therapy, and might influence the biodistribution of recoiled daughters and therefore increase the efficacy of the treatment. When the Ac-225 radiopharmaceutical is internalized, it is hypothesized that the recoiling daughters remain within the cell, reducing the potential non-targeted adverse effects (Ruigrok 2022; Ruigrok et al. 2019).

Based upon the choice of biological vector and mechanism of action, the dose can be calculated for the targeted and the non-targeted tissues (Handula et al. 2023). Here the high LET energy deposition influences the biological effects, and was confirmed by radiobiological modeling (Handula et al. 2023). While double-stranded DNA breaks are crucial for optimal treatment, it's important to acknowledge that high linear energy transfer (LET) also induces indirect effects and bystander effects, contributing significantly to the efficacy of the therapy (Widel 2017). Ruigrok et al. compared Lu-177 versus Ac-225 labelled PSMA and zoomed in on the differences in (radio-)biological effects and showed an increase of double strand DNA breaks while using Ac-225 in comparison to Lu-177 (Ruigrok 2022).

### Radiolabelling

In the case of Ac-225, achieving a therapeutic effect in both in vitro and in vivo experiments requires only kilobecquerels (kBq) of activity, this contrasts with the megabecquerels (MBq) of activity needed for commonly used beta-radiopharmaceuticals (Lu-177, Y-90 and Tb-161) (Blois et al. 2012; Hooijman et al. 2022).

When optimizing the conditions of the radiolabelling especially towards clinical implementation (Table 2), the use of a downscaled labelling is recommended, thereby using a reduced amount of activity in the same concentration in terms of molar activity, and final quencher concentration should be maintained. In this case not only the handling of activity is reduced, but it will also minimize the waste and costs. De Zanger et al. already proved that for Lu-177 downscale model could be applied (Blois et al. 2014; Zanger et al. 2019).

**Table 2:** General overview of important aspects which can be applied when performing a Ac-225 labelling

	Addition of quencher(s), dependent on the molecule (Blois et al. 2012; Blois et al. 2014; Larenkov et al. 2023)
	Ex. ascorbic acid/ascorbate/gentisic acid/ethanol/methionine/cysteine (0.35–50 mM*)
	Addition of buffer(s), pH, and molecule dependent
	Sodium-Acetate (pH 5, 0.1 M*) (Pretze et al. 2021), TRIS (pH 9, 14 mM*) (Hooijman et al. 2021)
	Add a titrated and a biological vector of high chemical purity to be able to obtain the required molar activity (Breeman et al. 2014) and mix carefully. Ensure the absence of metals within the used chemicals and solutions
	After dissolving the activity, direct use is recommended to avoid increase of impurities. Addition of the activity should be done as the last step before the labelling. Be aware of the impurities that might be released by the vial due to increased exposure
	Preferred final volume depending on the application (~ 500 kBq/140 µL preclinical (Handula et al. 2023) and ~ 10 MBq/0.5–1 mL for a clinical radiolabelling (Hooijman et al. 2021)), note that the smaller the volume, the better the kinetics, large volumes show low labelling yield, smaller volumes show an significant increase of radiolysis
	Optimize conditions → Ex. time and temperature per biological vector
	Rapid stabilization with an excess of quencher directly after heating (> 3 × as during labelling*)
	Because of the small volume, radiation in the sample is high, therefore, rapid stabilization should be performed
	Excess of DTPA/EDTA (> 10^4^ × more versus Ac-225) to complex all free Ac-225 and recoiled daughters to ensure rapid excretion (Kratochwil et al. 2017)

*Based on the final concentration

For each labelling step similar aspects should be considered as for labelling with radiometals. An overview of important notes for radiolabelling with Ac-225 is given in Table 2. Standardized amounts cannot be given as they must be optimized for each radiopharmaceutical specifically.

Despite the low molar activities for Ac-225 labelled radiopharmaceuticals (10–200 kBq/nmol), there are still difficulties to obtain high labelling yields, which is dependent on the compound, chelator, heating time, temperature, pH, volume and (quencher) concentrations within the solution. Based on the relative high toxicity of Ac-225, the degradation of the radiopharmaceutical should not be underestimated, as low amounts of radioactive impurities may already result in lower efficacy and possibly toxic effects. Depending on the labelling kinetics, different scavengers and the concentration thereof must be optimized accordingly (Blois et al. 2012).

### Quenchers

As described by Larenkov et al. (2023), radiolysis occurs in aqueous solution caused by the radicals formed by the interaction with water (Hooijman et al. 2022; Houée-levin 2002). To maintain high purity of the labelled biological vector and enhanced shelf-life, radiolysis have to be reduced by addition of radical scavengers (Larenkov et al. 2023). It is unknown whether radiolysis caused by the decay and emission of alpha particles itself (helium-nuclei) can be protected or blocked using any radical scavenger/quenchers. However, to reduce the subsequent formation of other radicals, different quenchers can be used (Shubin and Dolin 1963). Quenchers like ascorbic acid, cysteine, gentisic acid and ethanol, have been investigated for the stabilization of Lu-177 and In-111 labelled radiopharmaceuticals (Blois et al. 2012; Blois et al. 2014; Larenkov et al. 2023) and have shown to be useful for the stabilization of Ac-225 as well (Hooijman et al. 2021; Miederer 2022; McDevitt et al. 2002; Robertson et al. 2018b). High concentrations of quenchers (0.35–50 mM) are required to stabilize therapeutic radiopharmaceuticals however, currently, only a relatively low stability in time can be established (RCP > 90%) (Hooijman et al. 2021).

## Quality control (QC) and detection

Various aspects should be considered when performing quality control of Ac-225 labelled radiopharmaceuticals. Alpha particles are difficult to detect with standard detectors which are implemented in regular lab equipment such as radio-TLC, HPLC, etc. Therefore, Ac-225 should be in equilibrium with the daughter radionuclides before using quantification based upon Fr-221, that can be detected with common lab equipment.

The overall quality and thus the release of radiopharmaceuticals for patient use is dependent on parameters like the radiochemical yield (RCY) and the radiochemical purity (RCP). The RCY is defined as the ratio (%) between the labelled radiopharmaceutical and/or free chelate-complexed activity. The RCP is defined as the ratio between intact radiopharmaceutical versus other radioactive components present, including the radiolysis components (Coenen et al. 2017). For each QC technique, many additional parameters must be considered like the geometry, sample size, lower level of detection (LOD) and lower level of quantification (LOQ). Furthermore, cross-calibration between used equipment for QC is recommended to ensure the final quality of the radiopharmaceutical, therefore, a calibration source is required.

Since alpha radionuclides are highly toxic; the health physics regulations are adjusted accordingly by the national or local authorities and therefore limiting activity handling. For example, within the Erasmus MC (Netherlands), only amounts in the kBq range (~ 10 kBq) of Ac-225 are allowed to be handled for QC measurements in a regular class lab outside of the fume hood. Additionally, at least 1% of the impurity should be detected when performing the QC. As a result, sensitive measuring techniques and equipment are required to be able to detect very low quantities (< 0.1 kBq).

### Equilibrium

Within the decay chain of Ac-225, gamma emissions are generated from Fr-221 and Bi-213 decay (Fig. 1). A secular equilibrium develops after 6 half-lives of related daughter(s), meaning an equilibrium is formed between the Ac-225 and Fr-221/Bi-213.

The measurements of Ac-225 are based on the detection of gammas, either Fr-221 detection (t^1^/_2_ 4.8 min, 218 keV) or Bi-213 detection (t^1^/_2_ 45.6 min, 440 keV) may be used. Considering the current stability that can be obtained (as previously mentioned) with Ac-225 labelled radiopharmaceuticals, the detection with Fr-221 provides a more efficient and timely approach for quality control, as the onset of the Bi-213 equilibrium (ingrowth of 98%) sets in after 4.5 h, which takes too much time for releasing the Ac-225 labelled radiopharmaceuticals for patient use in the current methods. It can be acknowledged that solving these stability concerns may overcome the challenge posed by the Bi-213 detection time frame.

After performing any separation method used for i.e. radio-TLC and HPLC, a new equilibrium must be formed before starting the measurements of quantification. To avoid an underestimated outcome in the RCY or RCP, this requires a minimum of six half-lives (± 98% ingrowth) for Fr-221, (~ 30 min) and preferably later time point measurements for an increased ingrowth of the daughter nuclide (Hooijman et al. 2022).

**Fig. 4:**
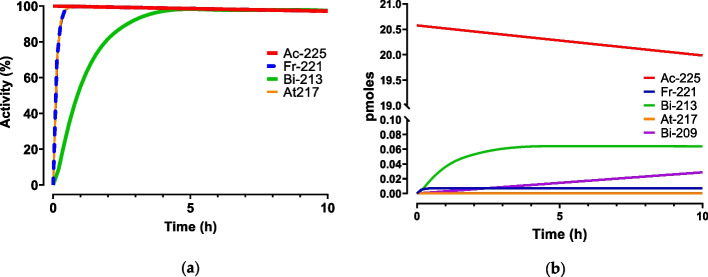
Ingrowth of the radioactivity of the daughter nuclides of Ac-225 in percentage of the total activity (%) (**a**) ingrowth of the daughters in moles, calculated for a 10 MBq source of Ac-225 over time including the ingrowth of the stable decay product Bi-209 (**b**)

Figure 4 gives an overview of the ingrowth of the different daughter (radio-)nuclides of Ac-225 and related concentration of the specific daughter (radio-)nuclides in picomoles, over time. In comparison to the regularly used beta-radionuclide therapy (Lu-177), the molar activity is approximately 600 × higher than for Ac-225. The influence of the ingrowing daughter nuclides can be neglected and will not have a significant impact on the labelling kinetics. On the contrary, competition with metal ions does play a major role.

**Fig. 5:**
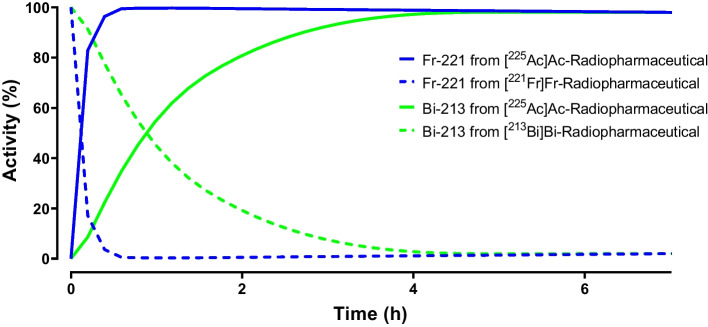
Presence of Fr-221 and Bi-213 in different chemical forms after labelling of [^225^Ac]Ac-PSMA-I&T

When Ac-225 and other alpha daughters’ decay, the energy that is released upon decay can reach a higher level than the binding energy of the corresponding chemical bonds. This leads to the release of the radionuclide out of the chelator of the radiopharmaceutical; this phenomenon is called recoil, as described earlier (Nelson et al. 2020; Kruijff et al. 2019). Additionally, different chemical forms are present, as shown in Fig. 5*,* underlining the importance for the onset of the equilibrium after > 30 min when performing measurements in specific energy windows. Algorithms are available for adapting for the ingrowth of the equilibrium; however, these methods do not consider the different chemical forms that are present (Castillo Seoane et al. 2022).

To perform accurate measurements of the Ac-225 labelled radiopharmaceutical, indirect measurements are required to obtain a representative outcome, due to the presence of recoil daughters in the sample which are also partially labelled (here Fr-221/Bi-213). Unfortunately, up until now, there are limitations for direct and accurate measurements of the alpha- ‘s for Ac-225 radiopharmaceuticals. Since multiple alpha daughters are present upon Ac-225 decay, with minor differences in energy (Table 2), radio-TLC and HPLC analysis is particularly difficult, also considering high throughput measurements (“HP-Ge detector” section).

### Systems and settings

Due to the relatively high impact of alpha radiopharmaceuticals and the difficulties related to detection, for validation of a Ac-225 radiopharmaceutical multiple techniques should be used. The RCY can be measured by performing ITLC separation, which can subsequently be quantified by the radio-TLC scanner (Thakral et al. 2021), gamma counter or HP-Ge detector. The RCP can be determined by measuring the collected HPLC fractions in the gamma counter for indirect measurements (Hooijman et al. 2021).

The outcome of these measurements is dependent on the used hardware and software, sensitivity, and the resolution of the used detectors. The sensitivity of the detectors is of significant importance since the activity in the samples is limited due to health physics regulations. Due to the challenges of QC analysis, cross-validation of the different methods is recommended to ensure the quality (Bergeron et al. 2022).

#### Dose calibrator

For direct measurements, a dose calibrator can be used, only when the solutions are in equilibrium. As the amount of activity (MBq) is low, the accuracy of the dose calibrator is of crucial importance. Validation was performed at our institute with an adapted setting from the Tc-99 m channel with an adaptive factor (factor = 148), however, this is dependent on factors like the type of system and the corresponding geometry (Hooijman et al. 2021).

#### Radio-TLC analysis

For separation of the chemical forms of the radiopharmaceutical, ITLC strips are utilized with specific eluents. Following the chemical separation process, the radio-TLC scanner can accurately identify and measure non-incorporated radionuclide, complexed with chelate (for example DTPA), and labelled radiopharmaceuticals (Salvanou et al. 2020). An essential advantage of the radio-TLC scanner is that it is quick and resistant to recovery issues.

Due to the low activity concentration of Ac-225 in a preclinical setting, it will be a bigger challenge to set the parameters for ITLC separation, this must be considered in advance. To ensure the RCY, crucial measurements with the radio-TLC include determining the LOD, LOQ along with the linearity within the low activity range. For clinical applications, at least 1% of impurities should be detected. i.e. from a QC sample (~ 5 µL/50 kBq at the Erasmus MC), and the LOD/LOQ should be evaluated accordingly (< 500 Bq) (EDQM 2022).

The detection crystal size and width of the detector are of importance for obtaining sufficient count and thus statistically significant results. Previously conducted research made use of a NaI(Tl) scintillator detector, with a 2.54 × 2.54 cm crystal size, and digital multichannel analyzer (MCA) to perform the RCY measurements. Different speeds of scanning were evaluated (0.2–0.6 cm/min), where 0.6 cm/min, from the bottom to the top of the ITLC strip, resulted in a total scanning time of 900 s with a total amount of counts of > 20.000 cps, < 1% error (Hooijman, et al. 2021). The energy resolution (KeV) of the radio-TLC detector must be sufficient for measuring the energies of Fr-221 (218 keV) and Bi-213 (440 keV) separately after the onset of the equilibrium. An important note is that a phosphor imager does not suffice, as this device most often does not have the ability to separate the different energies and therefore such a system is to our opinion not sufficient and should not be used QC for Ac-225 containing radiopharmaceuticals.

Consequently, it is recommended to evaluate multiple mobile phases to obtain separation between the unlabelled Ac-225, [^225^Ac]Ac-chelate, and [^225^Ac]Ac-radiopharmaceutical. Solutions of sodium citrate or an acetonitrile/water combination, while varying molarities and pH is often used. In practice, for Ac-225 radiopharmaceuticals, higher concentrations of sodium citrate (0.5–1.0 M) show an increased peak resolution (Hooijman et al. 2021). The separation parameters, based upon the radio-TLC profile should be set and validated.

#### Gamma counter

A gamma counter (i.e. a thallium activated, sodium iodide crystal) should be calibrated for Fr-221 (and Bi-213, if required) specifically in an appropriate region of interest (ROI). Also, here an onset of an equilibrium is required. It is important to note that the detection of Fr-221 can be influenced by the backscatter of Bi-213 causing additional counts within the energy windows. A backscatter correction can be applied using dedicated software. To ensure RCY, as for all other equipment used, it is important that the LOD, LOQ, and linearity in the low activity range can be measured accurately. Additionally, quantification of the activity concentration is used in our institute to verify the amount of activity. Furthermore, to be able to perform accurate calculations, the geometry of the samples that are measured should be standardized. The gamma counter is overall more sensitive than the radio-TLC-scanner, with a high precision and accuracy. For high sample throughput measurements, the use of the gamma counter is recommended.

**Fig. 6:**
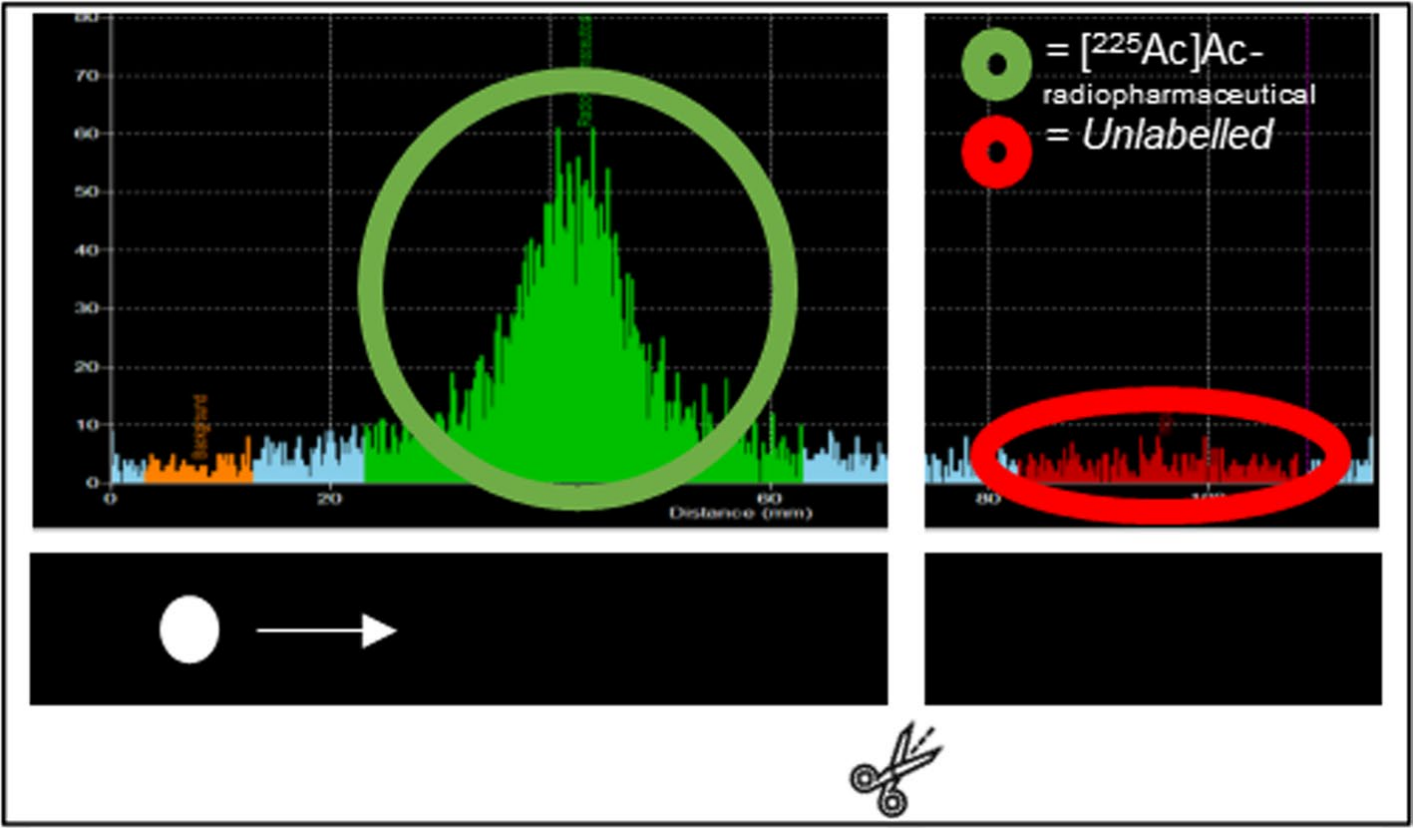
Example of separation profile of labelled and unlabelled Ac-225-radiopharmaceutical

For the cross validation of the RCY measurements, the ITLC-strip used for TLC-scanner can be cut at the appropriate retention factor, after separation is proven between the labelled and unlabelled radiopharmaceutical (Fig. 6). Thereby the top and the bottom of the strip can separately be measured in the gamma counter or HP-Ge detector (“HP-Ge detector” section), thereby also enabling the calculation of the RCY as the ratio between the top and bottom of the ITLC strip. Additionally, all collected fractions from HPLC analyses for indirect measurement can be measured in the gamma counter as well, see “Radio-HPLC analysis” section.

#### HP-Ge detector

For measurements with a high energy resolution (< 1.2 keV @ 122 keV and < 1.8 keV @ 1332 keV), the use of the HP-Ge detector is preferred, as there is less influence of backscatter. Due to the flat detector geometry, a loss of counts must be considered, leading to a lower sensitivity and higher need for geometry correction. Although the HP-Ge detector has a low throughput, performed measurements will result in a more accurate output in comparison to radio-TLC and gamma counter measurements.

Measurements for radionuclidic purity, nuclide identification and RCY can be performed with the HP-Ge detector. For measurements and quantification, a multinuclide source is required for calibration within the appropriate energy range. The HP-Ge detector should be calibrated for the geometry and measuring time. The radionuclidic purity is dependent on the Ac-225 production method which can lead to the presence of Ac-227, U-233, Th-229, and/or Ra-225.

Consequently, samples should be measured after ~ 190 d, to let all the Ac-225 decay for measurement of the present Th-229 (99 keV (0.26%), 102 keV (0.81%), 136 keV (1.18%), 156 keV (1.19%), 193 keV (4.41%), 210 keV (2.80%)). For quantification, also the presence of Fr-221 and Bi-213 can be used. The amount of Th-229 should be represented as ppm per final Ac-225 activity. For Ra-225 contamination (40 keV, 30% yield) can be measured after ~ 12 h, if no Ra-225 present, minimal detectable activity (MDA) should be used.

#### Radio-HPLC analysis

As previous research shows the limited detection of degradation products with ITLC separation, implementation of an appropriate HPLC-method is required (Hooijman et al. 2021, 2022; Larenkov et al. 2023). A HPLC-method should be chosen based upon a gradient and a column that can separate all the related impurities, and should be validated based upon the most recent EANM guidelines (Hooijman et al. 2021; Gillings et al. 2021).

**Fig. 7:**
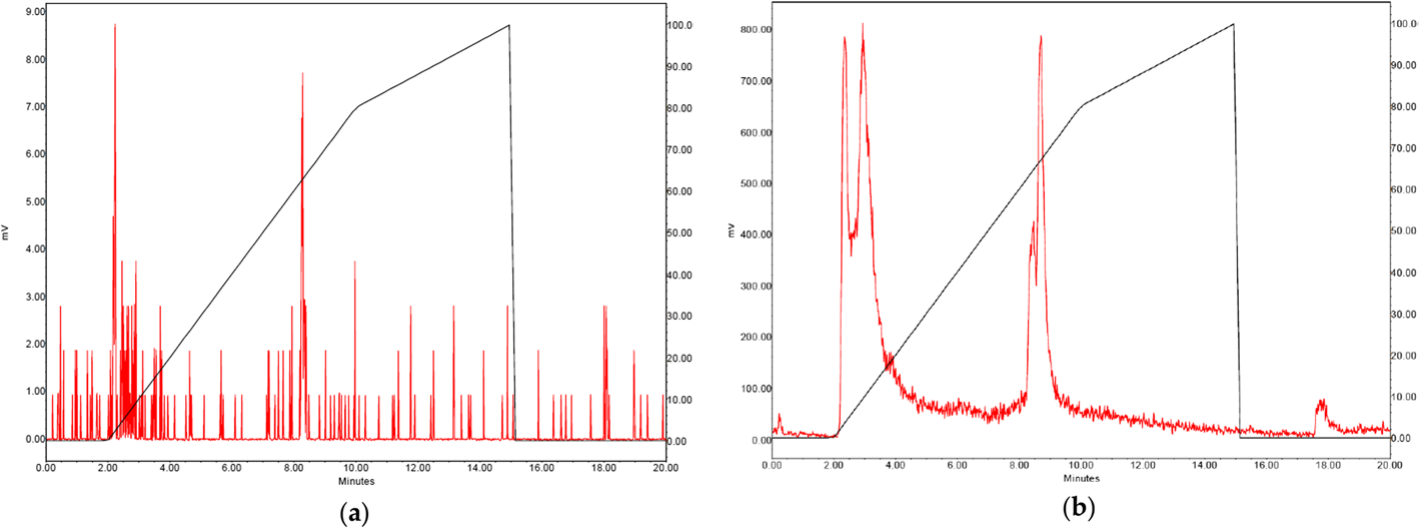
Gamma detection (**a**) and liquid scintillation (**b**) of Ac-225 from different chemical forms after injection of [^225^Ac]Ac-PSMA-I&T (method Fig. 9)

Following the HPLC-method setup, direct measurements were performed on HPLC coupled to an inline sodium iodine gamma detector (1-inch NaI(TI) Scionic crystal (Bunnik, The Netherlands) connected to a Canberra Osprey multichannel analyzer and signal amplifier (Zellik, Belgium), Fig. 7a) and/or liquid scintillation detector (Beta-ram radio-HPLC detector (Lablogic Chantilly, USA), Fig. 7b). As shown in Fig. 7a, a too low signal was observed when 10 kBq was injected. However, when using the liquid scintillation method, a higher sensitivity was observed. Unfortunately, the interpretation is difficult, due to the recoil and subsequent different present chemical forms (Fig. 5). To our knowledge, there is currently no alternative for direct measurement of Ac-225/Fr-221 on a HPLC (Hooijman et al. 2021).

**Fig. 8:**
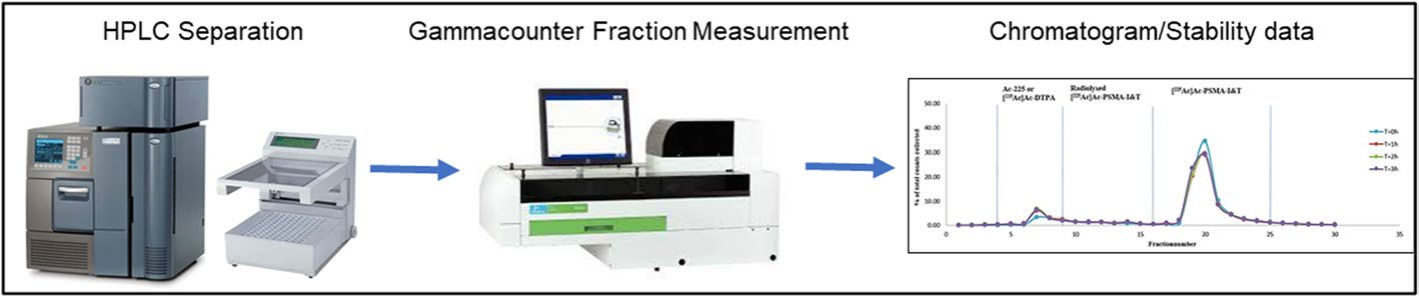
Flow diagram of indirect measurement for Ac-225 labelled radiopharmaceuticals

In the clinical quality control setting, indirect measurement must be implemented; measuring gamma emissions of the daughter radionuclides once an equilibrium has been established. The eluate has to be collected after injection by a fraction collector (set appropriate volume and time, for example 0.5 mL/min) and the collected fractions should be measured in the gamma counter (Fig. 8).

**Fig. 9:**
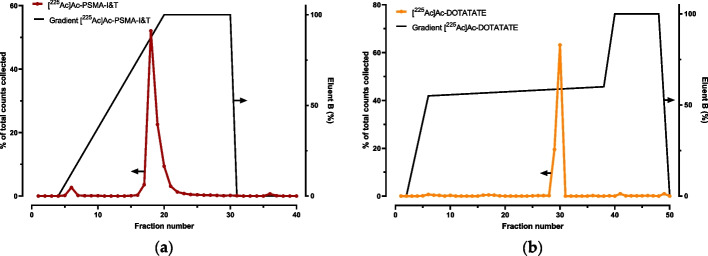
Example of a chromatogram generated by plotting Fr-221 counts obtained from samples collected by a fraction collector after HPLC separation (LiChrospher RP-18 endcapped (5 µm)) Merck, Amsterdam, The Netherlands) for [^225^Ac]Ac-PSMA-I&T (Eluents A: Water containing 0.1% TFA and 5% acetonitrile, B: acetonitrile containing 0.1% TFA and 5% water), gradient 0–2 min 100% solvent A, 2–10 min to 100% B, 10–15 100% solvent B, 15–20 100% solvent A) (**a**) and [^225^Ac]Ac-DOTA-TATE (Eluents A: 0.1% TFA, B: MeOH, gradient 0–2 min 100% A, 3–20 min 45–40% A, 20–25 min 100% B, 25–30 min 100% A) (**b**)

Counts and tube numbers must be plotted by fraction number to obtain a chromatogram, which is required for the calculation of the RCP as shown in Fig. 9 for [^225^Ac]Ac-PSMA-I&T and [^225^Ac]Ac-DOTA-TATE.

Since the indirect HPLC analysis is time-consuming and the relative short shelf-life of the Ac-225 labelled radiopharmaceuticals, it is a major challenge to include the RCP as part of the release criteria and has to be considered to be determined retrospectively. Therefore, the HPLC including fraction collector for determination of RCP and stability should be a part of the validation phase. It must be noted that during this phase of validation, the recovery has shown to be an important parameter to consider.

## Preclinical use of Ac-225

Currently, due to the limited supply of Ac-225, often only small amounts of Ac-225 are available and mainly preclinical in vitro and in vivo experiments are performed (Ruigrok 2022; Handula et al. 2023; Kratochwil et al. 2020a; Banerjee et al. 2021; Kelly et al. 2019; Tafreshi et al. 2021; Müller et al. 2014; Umbricht et al. 2019; Busslinger et al. 2022). When using Ac-225 in a preclinical setting, lower amounts (kBq’s) are used for synthesis and QC in comparison to in a clinical setting (MBq). Therefore, precautions regarding the activity handling must be taken, since measurements could be more near the LOD or the LOQ. Due to the low quantities, the used equipment should be well calibrated in the lower ranges by also considering the related equilibrium between daughter nuclides.

Depending on the local regulations and health physics authorities, a regular fume hood including filtering system in a dedicated lab would be sufficient (labelling ~ 1 MBq), but a glovebox is still preferred. A dedicated hood for handling Ac-225 only is recommended to decrease the contamination risk, including reducing the potential cross-contamination with other commonly used radionuclides. Reducing the contamination risk is of significant importance, especially when radionuclides containing similar energies (In-111, 245 keV, Lu-177, 208 keV) are present, and interfere with Ac-225 related measurements. To the best of our knowledge, as of the current writing, there are limited (pre-)clinical studies published that provide comprehensive practical details on the use of Ac-225. The details presented here are based on our own experiences.

During sample quantification, it is crucial to consider the equilibrium between Ac-225 and Fr-21/Bi-213, to avoid potential false-positive or false-negative outcomes. This precaution ensures that the samples are representative for using the (Fr-221/Bi-213) indirect measurement technique. Practical considerations are for in vitro experiments include that after each washing of cells a new equilibrium should be established. When performing an animal study, quantification of the injected Ac-225 labelled radiopharmaceutical is a challenge since empty syringes can only be measured in a gamma counter or HP-Ge detector to correct for the remaining activity. Furthermore, when performing in vivo experiments, it is recommended that mice are inhabited in isolation, to reduce the possible contamination risk.

## Clinical Ac-225-labelled radiopharmaceuticals

### Radiation protection and health physics

As described previously, alpha-emitting radionuclides are known for their high energy emission and high toxicity, which is especially important when working with clinical quantities. Firstly, to minimize the risks, a radiation risk assessment is required for the safety of the staff who perform the activity handling.

Due to the toxicity and half-life of Ac-225, low activities (MBq) are required to prepare a therapeutically dose, in comparison to beta^−^ isotopes (GBq). Small contaminations on either analysis systems or working space with Ac-225 may have a major impact. Additionally, it is recommended to measure the used surfaces and equipment by taking wipe tests to be able to measure low counts. Measurement with an alpha probe is recommended as it can very efficiently detect small quantities of alpha particles.

Analysis systems are often placed outside a fume hood, and therefore lower quantities of activity can be injected. As compared previously, for the extensively used β- isotopes, this is often in the MBq range, whereas for Ac-225 radiopharmaceuticals, this is in the kBq range. As widely used radioisotopes such as Lu-177 and In-111 have energies within the same energy windows (200–300 keV) contamination from any previous injections have a substantial influence as a carry-over of 1% can thereby have a major influence, therefore, a dedicated HPLC system is recommended.

Additionally, according to the Ph. Eur. guidelines, the production of radiopharmaceuticals and QC should not be performed in the same space. As strict health physics regulations apply, this may be especially difficult and must be discussed up front.

At the time of writing, limited information is published on the clinical procedures when working with Ac-225 and the practical details are lacking. The context for Ac-225 handling depends on the local regulatory guidelines, radiation safety protocols, risk assessments, facility, and training. Therefore, details based on own experience are shared.

### Preparation of clinical Ac-225 labelled radiopharmaceuticals

#### Starting materials

Currently, there is no GMP quality Ac-225 available and the radiopharmaceutical production for studies will therefore often involves the use of the non-GMP Ac-225, GMP-grade pharmaceuticals and solutions. Additionally, there is also no non-radioactive reference isotope for actinium available to be use for labelling as a UV standard. Consequently, it is important that the (radio-)chemical precursor or peptide are GMP-compliant and should be supplied sterilized and assessed on purity, especially on the presence of metal ions. To prevent a low RCY, the used labelling procedures should be assessed in advance on the influence of the potential metal contaminations and must be performed metal free to avoid interference during the reaction.

Due to radiation safety regulations and to decrease the risk of contaminations with the relative high radioactivity for clinical purposes, it is recommended to use a suitable closed system for heating. Within our institute, a Biotage microwave (Biotage Initiator+, Biotage, Uppsala, Sweden) was used with specific vials designed to stand a pressure of 30 bars. Unfortunately, the dedicated vials are non-GMP and to be able to work sterile and metal free, cleaning and sterilization (25 kGy) of these vials is required (Nguyen et al. 2007).

Even though the Ac-225 stock (non-GMP) and related half-life (9.9 days) allows for the distribution from the production sites, there is no set expiration date for the use of the stock solution. As stated earlier, impurities are formed in the Ac-225 stock vial, due to the interaction between the Ac-225 and the vial. Based upon our own experience, the solution could be used up to two weeks after dilution, thereafter the RCY reduces drastically.

#### Radiolabelling

As a consequence of the toxicity of Ac-225 and the related daughters, and the amount needed for the preparation of a patient dose (> 10 MBq) in comparison to preclinical work (< 1 MBq), it is advised that Ac-225 should exclusively be handled in a closed system (for example a hot cell or isolator). For alpha emitters like Ac-225, which have only relative low yields of gamma energy co-emission, working in glovebox is sufficient as the risk is not based on proper shielding but on the risk of contamination.

Study based radiopharmaceuticals have to be labelled in a GMP setting, where aseptic handling and/or preparation, must be performed in a Class A glovebox including a locker system (Class B), thereby the background in the room should be Class C. Dedicated shielding of the Ac-225 sources in the glovebox and related waste management should be considered.

As mentioned previously, to reduce the contamination and increase the robustness of the radiolabelling, the labelling can be performed with a temperature-controlled microwave instead of a heating block. By manual preparation, only a glovebox would be sufficient. A microwave should preferably be installed in a separate cabinet, as disruption of airflow increases the risk of biological contamination of the product.

Based on our experiments, high levels of radiolysis are formed during the heating step, underlining the necessity of using quenchers during labelling. As a result, a RCP of > 90% was obtained by decreasing the temperature and the heating time, and by decreasing the concentration of the activity (MBq/mL). An advantage was observed by using a microwave for heating including rapid cooling over the use of a heating block. To stabilize a solution of Ac-225 labelled radiopharmaceutical in time, an excess of quencher directly after labelling showed an improvement in the stability without interfering with the labelling kinetics (Hooijman et al. 2021).

**Fig. 10:**
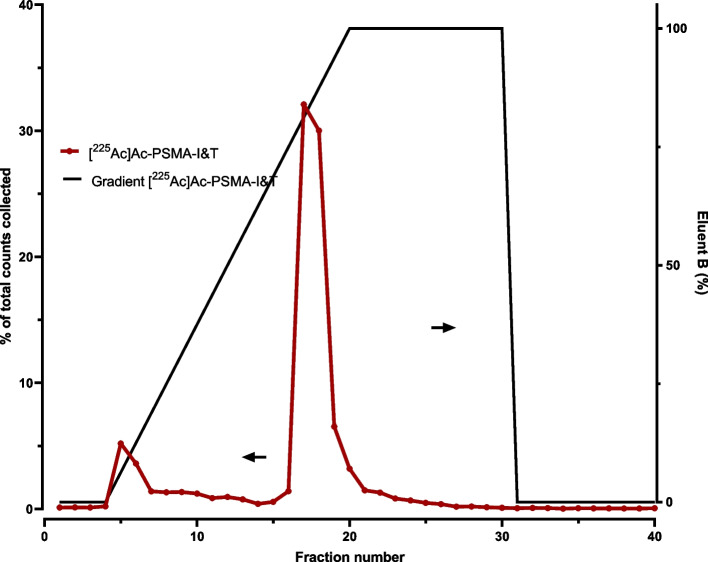
Labelling with a high RCY was injected onto the HPLC (**a**) and a chromatogram of [^225^Ac]Ac-PSMA-I&T was obtained, based on indirect Fr-221 measurements. A low RCP labelling (< 60%), showing radiolysis in fractions 5–15. The chromatogram was identical after labelling was SPE Seppak purified and reinjected, confirmed that the peak at fraction 5–15 is still there and thus contain an Ac-225 labelled radiolysed product (**b**)

The presence of alpha particles shows a relation with the introduction of a peak which appears directly after elution of the dead volume of the HPLC system in overlay with unlabelled peak, indicating a specific type of radiolysis, for now only confirmed for [^225^Ac]Ac-PSMA-I&T and [^225^Ac]Ac-DOTA-TATE (Hooijman et al. 2021) (Fig. 10). This profile is different in comparison to other radiopharmaceuticals labelled with isotopes such as Lu-177/In-111 (Blois et al. 2014). The mechanism for this type of damage must be further investigated. Unfortunately, due to the low mass of the radiopharmaceutical used, and the high related radioactivity, it is a challenge to qualify the related fragments. In our situation, a Sep-Pak C_18_ purification was used to differentiate between free radionuclide, DTPA-complexed and radiolysed radiopharmaceutical. Thereby, reinjection after purification confirmed that the impurity is labelled radiolysed radiopharmaceutical.

If the labelling solution is diluted for clinical administration, it is important to note that the quencher concentration is also decreasing, therefore, the same quencher concentration should be maintained after dilution for stability purposes. Consequently, an increased activity concentration might have a positive effect on labelling kinetics and thus the RCY.

#### Labelling methods

There are different methods described for the production of Ac-225 labelled radiopharmaceuticals; Manual production (Hooijman et al. 2021; Yang et al. 2022; Thiele et al. 2017; McDevitt et al. 2001; Kratochwil et al. 2020a; Tafreshi et al. 2019), semi-automated production and fully automated production (Pretze et al. 2021). For the radiolabelling, practical aspects have to be considered, as described in Table 3.

**Table 3:** General overview and practical considerations for the radiolabelling with Ac-225

Step	Critical handling
Preparation of the equipment and solutions	Metal free handling
Measurement of the Ac-225 stock	Validated geometry
Labelling	Order of addition, never add activity to pharmaceutical before addition of the quencher Excess DTPA after labelling (DOTA-chelate)
Stabilization	Rapid stabilization with quencher after heating
Quality control	When the time after separation increases, the accuracy of output increases
Filtration	Loss of activity

Currently, the labelling is mostly conducted in a manual way as small volumes are used. An increasing demand for the Ac-225 labelled radiopharmaceutical may favor the use of a (semi-)automated method or a kit-based method over a manual method, especially concerning the health physics regulations.

#### Validation

When working with alpha-emitters, it should be noted that this has a substantial impact on the infrastructure and equipment, especially regarding the validation of the QC techniques and the sterility of the Ac-225 labelled radiopharmaceuticals. The validation of the (QC) equipment should be performed according to the most recent EANM guidelines (Gillings et al. 2020, 2021, 2022). A prospective risk analysis has to be performed, and subsequent validation according to the prescribed parameters has to be conducted for the dose calibrator, gamma counter, radio-TLC scanner, HP-Ge detector and HPLC. Difficulties and challenges regarding Ac-225 measurements are described in “Systems and settings” section. Conclusively, separation techniques for the unlabelled and labelled radiopharmaceutical are described per detection system and have to be validated according to the appropriate guidelines. In our experience, special attention has to be given to radionuclidic purity of the non-GMP Ac-225 and the determination of RCP including recovery for the HPLC measurements which are both time-consuming processes (*see * “Systems and settings” section).

#### Sterility

Generally, radiopharmaceuticals are produced as a sterile aqueous solution. The microbiological contamination should be evaluated by an endotoxin test (< 5.0 EU/mL) and the aseptic manufacturing process needs to be validated with a media fill and bioburden. To test the sterility in the final production vail, ~200 days of waiting time is required for decay, to conform to the radio safety measures that apply for the release of the microbiological samples. To our opinion, the outcome of these measurements is doubtful and the relevance of this test for Ac-225 labelled pharmaceutical has to be considered. Sterile starting materials and chemicals must be used to ensure the sterility of the end product. The used (microwave) vials for labelling were only available as non GMP-grade, and should therefore be sterilized by gamma radiation (25 kGy) as described previously. As a non-GMP Ac-225 source is used, sterility is not guaranteed. Dose calculations were performed in house for the Ac-225 stock solution and based on calculations, the solution may be considered sterile as the dose is 98.2 kGy/h (30 MBq/30µL, 0,03 cm^3^ water, IDAC dose Ac-225/daughter nuclides), resulting in > 25 kGy in 15 min (Nguyen et al. 2007; Andersson et al. 2017).

#### Release criteria

Before implementing the Ac-225 radiopharmaceutical, it is required to have product specifications. Table 4 shows an example of release criteria that can be set for a patient batch.

**Table 4:** Example release criteria clinical patient batch

Parameter	Quality control technique	Release criteria (based upon literature)
*Preliminary release*		
Clear solution?	Visually	No particles visible
RCY	Radio-TLC Gamma counter HP-Ge detector	> 95%
Radionuclide identification	HP-Ge detector	Presence Fr-221/Bi-213
pH	Indicator paper	Between 5.5 and 9
Final activity	Dose calibrator Gamma counter	Patient dose ± 5%
Endotoxins	Endosafe PTS system	< 5 EU/mL
Filter integrity	Filter integrity test	Passed
*Final release*		
RCP	HPLC/fraction measurement (gamma counter)	> 90%* up to 3 h

*In the risk-analysis, the RCP is discussed and resulted in a release criterion of the HPLC of 90%, here the risk of the 5% decrease was accepted, based on toxicity calculations and distribution of unlabelled Ac-225 is based on; **1**. The unbound Ac-225 is complexed for 25% with DTPA and excreted by the kidneys (ICRP 2015). **2**. 25% remains unbound (ICRP-30) and **3**. 50% will be bound to PSMA metabolites (ICRP128 model for F-18 amino acids)

For the radioactivity that is injected, a dose of < 1% unlabelled radionuclide may already have a big impact, this is a relatively high dose that will distribute to the bones mainly. As the free radionuclide binds to the DTPA that is added in excess, the DTPA-complexed (daughter-)nuclide will directly be excreted by the kidneys (Capello et al. 2003). Therefore, it is recommended to add an excess of DTPA after radiolabelling. Before release of the radiopharmaceutical, final releasing documentation should indicate that all prospective testing has been completed (preliminary or final).

## Clinical targeted alpha therapy

At the moment of writing, formal phase I studies according full GMP with Ac-225 labelled radiopharmaceuticals are rare. Most of the studies are based on (small) retrospective studies (Ling, et al. 2022; Morgenstern et al. 2018, 2020). In this chapter, some practical aspects of the clinical application related to the use of Ac-225 are summarized.

### Clinical administration

In the literature, limited information is available on the practical aspects regarding clinical administration. For example, high concentrations of quenchers are required to stabilize Ac-225 labelled radiopharmaceuticals, and therefore the osmolarity might be exceedingly high (> 2000 mOsmol). To compensate for the high osmolarity, the radiopharmaceutical should be administered simultaneously with a saline solution. For example, [^225^Ac]Ac-PSMA-I&T was administered simultaneously using two separate infusion pump systems with a 1:4 ratio (Hooijman et al. 2021). When starting the administration of [^225^Ac]Ac-PSMA-I&T, it is important to firstly start the infusion pump with saline to prevent infusion of high osmolarity solution. After the Ac-225 labelled radiopharmaceutical has been administered, the infusion lines should be flushed with saline to ensure complete administration. In conjugation with the technical aspects of the administration, it is important to take precautionary measures surrounding the patient administration itself. Due to the short range of Ac-225, there is no need for distancing during administration. The method of administration should be determined on beforehand, and personnel should be trained sufficiently. Leakage must be prevented by double-checking the administration system including the infusion lines and absorptive mats should be placed around the administration system. After the administration, all materials used for the administration should be collected in a radioactive waste container and be disposed.

### Biodistribution

The clinical biodistribution of Ac-225 radiopharmaceuticals is difficult to measure due to relatively low activity that is administered, and the imaging have to be based on the daughters (Fr-221/Bi-213). On top of that, when the recoil occurs, the radionuclide is also no longer complexed with chelator and might be present in the cellular matrix. The daughter radionuclides circulate and thereby follow the (nuclide-specific) distribution (Kratochwil et al. 2020a). It has been shown that early release of free Bi-213 causes an excess uptake in the kidneys of mice (Schwartz et al. 2011) and shows toxicity after 40 weeks (Jaggi et al. 2005). Up until now, there is no specific toxicity reported based on the daughter nuclides from Ac-225 labelled radiopharmaceuticals in patients.

For the assessment of the biodistribution, it is necessary to investigate the clearance by obtaining blood- and urine samples at different time points after administration. During the blood sampling it is important to avoid sampling from the same intravenous entry point in which the radiopharmaceutical is administered. Residual activity might remain in the intravenous entry point after administration, which could affect the results when measuring the blood samples. To prevent sampling error, a second intravenous entry point should be acquired. Furthermore, when measuring the blood- and urine samples it is also necessary to measure the activity of a reference sample. With the acquired data, time-activity curves can be calculated.

Ideally, imaging should be performed to determine the uptake in organs and to assess the recoil effect. However, it is important to know that only low doses are administered, and co-emissions might not be visible enough. Moreover, it is important to mentally prepare the patients for both the planning of the scan(s) and the scanning time (which is usually long for dosimetry purposes). By doing so, images with better quality could be acquired which subsequently leads to better dosimetry calculations.

Furthermore, due to the recoil of the daughter nuclides, delocalization throughout the body should be considered when performing biodistribution studies. An alternative approach may be to implement an element equivalent matched pair for imaging. Thereby, Ac-226 may be an option when working with Ac-225 to allow further investigation of the biodistribution and dosimetry, for example (Koniar et al. 2022). Currently, to our knowledge, this research is within the preliminary phase.

### Radioresistancy

Oxygen is one of the important players in the biological effects of radionuclide therapies as oxygen leads to more radical formation. Tumors with low oxygenation are more likely to be more resistant to ionizing radiation and therefore the effect of hypoxia needs to be considered when using low LET (beta-emitters) (Wulbrand et al. 2013). Hypoxia's impact is expected to be significantly reduced with high linear energy transfer (LET) due to the direct effect on the DNA. Furthermore, this is linked to the production of reactive oxygen species (ROS), leading to indirect toxic effects predominantly in non-hypoxic cells (Navalkissoor et al. 2022; Jiao et al. 2022). The advantages of using alpha-emitters after a limited effect with beta-emitters has been shown in several studies (Feuerecker et al. 2021). Even though remarkable benefits have been achieved with beta-emitters, part of the patients treated with beta-emitters still develop radioresistancy. As an example, approximately 30% of the patients treated with [^177^Lu]Lu-PSMA do not a show response or develop radioresistancy. For these patients, therapy combining alpha- and beta-emitters might be an alternative treatment option, thereby the tumor coverage is increased and the dependency on oxygen radicals decreases when using an alpha emitter. In prostate cancer patients, tandem therapy with [^177^Lu]Lu-PSMA combined with low-activity [^225^Ac]Ac-PSMA showed promising results (Khreish et al. 2020; Rosar et al. 2021; Satapathy et al. 2021; Kratochwil et al. 2020b; Privé et al. 2022a).

### Waste management

The (clinical) waste of Ac-225 theoretically can be discarded through the short-lived isotope waste. However, this is very dependent on the radionuclidic purity of the source and is related to the production pathway. Due to the number of used materials for QC and the collection of all fractions for indirect HPLC measurements, a relatively large amount of waste is produced. Additionally, as mentioned, depending on the production method, during the Ac-225 production or extraction, Th-229 and/or Ac-227 may be introduced in the batch and are long-lived isotopes. The radionuclidic purity per supplier should be investigated and quantified with either the HP-Ge detector or alpha spectroscopy. Due to the toxicity of Ac-225, waste should be handled with utmost care and inhalation or ingestion must be prevented, as a dose is mainly obtained through this route. Due to the characteristic’s, limited shielding is needed (Sgouros et al. 2019, 2021; Tollefson et al. 2021).

## Future prospective

### Availability of Ac-225

The availability of Ac-225 is a limiting factor for the further development of Ac-225 labelled radiopharmaceuticals. The current prospective is mainly focused on two supply chains. The current focus is on irradiation of Ra-226, facilitated by its availability, cyclotron production feasibility, and absence of Ac-227 occurrence. It is crucial to acknowledge, however, that the presence of radon gas during production necessitates specific handling protocols, and ongoing research is required to resolve this. In parallel, Th-229 is extracted from a nuclear waste sources to increase the availability and thereby the goal is to ensure a reliable Ac-225 supply (Yan 2020; Camacaro et al. 2023).

### Other potential alpha-emitters

Due to the limited availability of Ac-225, alternative alpha-emitters have been explored. Radionuclides such as Astatine-211, Bismuth-213, Terbium-149, Lead-212, Radium-223 or Thorium-227 are produced and are assessed by Eychenne et al. (2021) and provides an overview of not only the availability and chemical design, but also the preclinical and clinical data on these alpha-emitters. Use of alternative alpha emitters to Ac-225 with for example with a different half-life may be more effective in specific disease treatment strategies, as the dose delivered to the target tissue is increased. The preclinical and clinical data on alpha-emitters have recently been updated by Ling et al. (2022) and future prospective for the application of these alpha-emitters have been provided.

### Clinical implementation

The implementation is recommended to be performed with thoroughness due to the risks involved, thereby the quality of the Ac-225 labelled radiopharmaceutical that is produced, needs to be well established before it can be considered safe for clinical use. Hence, it's crucial to increase radiopharmaceutical production and enhance accessibility, recognizing the impact of existing stability challenges. Successfully resolving these issues could lead to centralized (GMP) production and distribution, marking a substantial step forward in the field towards implementation for clinical trials.

At this time, comprehensive clinical trial data with significant patient populations are scarce, while individual case reports are more prevalent (Morgenstern et al. 2018; Parida et al. 2023). Consequently, the need for meticulously planned clinical trials is emphasized, as extensively deliberated by Jang et al. (2023), describing numerous trials that are currently underway to explore the clinical effectiveness of mainly small molecules and antibodies radiolabelled with Ac-225. However, as described earlier, the effectiveness of the treatment requires careful consideration of both the physical and biological half-lives.

Furthermore, exploration of theranostic chelators for heat-sensitive molecules (e.g. antibodies) should be taken into consideration, as this further constraints the therapeutic potential of Ac-225. For example, Macropa is specifically designed for actinides and lanthanides, it should be noted that a new chelator is required to enable the theranostic approach (Blei et al. 2023). On the contrary, the DOTA-chelate can be used for diagnostic PET radionuclides (ex. Ga-68, Zr-89), but requires heating, and even higher temperatures are needed for radiolabeling with Zr-89 (Privé et al. 2022b), which makes it not suitable for heat-sensitive molecules. However, research on the safety and tolerability of both the types of molecules is still ongoing. Especially the need for quality control techniques is crucial, lacking adequate methods can have consequences for patient safety as suboptimal detection techniques may lead to potential side effects in the treatment. Ensuring accurate and reliable detection is crucial to guarantee the safety and well-being of the patient.

## Conclusion

Many challenges need to be overcome when working with Ac-225 labelled radiopharmaceuticals. Alpha particles are not only difficult to detect, but the high toxicity also influences the activity handling and QC analysis. To ensure the quality for clinical implementation, cross-validation should be performed with different QC techniques. Additionally, due to many health physics and methodological aspects that need to be considered, the process of producing GMP grade radiopharmaceuticals is time-consuming and requires experienced personnel. Exceptional benefits have been shown in patients treated with Ac-225 radionuclide therapy, which make this a remarkably interesting and encouraging treatment option to further investigate in well-designed clinical trials.

## Data Availability

The datasets used and/or analyzed during the current study are available from the corresponding author on reasonable request.

## Abbreviations

Ac-225: Actinium-225
Ac-226: Actinium-226
At-211: Astatine-211
Bi-209: Bismuth-209
Bi-213: Bismuth-213
Ci: Curie
DNA: Desoxyribonucleïnezuur
DOTA: Dodecane tetraacetic acid
DOTA-TATE: DOTA-0-Tyr3-Octreotate
DTPA: Diethylenetriaminepentaacetic acid
EANM: European Association of Nuclear Medicine
EDTA: Ethylenediaminetetraacetic acid
EOB: End of bombardment
Fr-221: Francium-221
GBq: Giga Becquerel
GMP: Good Manufacturing Practice
GRPP: Good Radio-Pharmacy Practice
Gy/h: Gray/hour
HCl: Hydrochloric acid
HEPA: High-efficiency particulate air
HP-Ge: High Purity Germanium (HPGe)
HPLC: High-performance liquid chromatography In-111: Indium-111
IPPE: Institute for Physics and Power Engineering
ITU: Institute for Transuranium Elements
kBq’s: Kilo Becquerel
KeV: Kilo electronvolt
kGy: Kilo Gray
LET: High linear energy transfer
LOD: Limit of detection
LOQ: Limit of quantification
Lu-177: Lutetium-177
MBq: Mega Becquerel
MeOH: Methanol
MeV: Mega-electron volt
Osmole: Osmolarity
ORNL: Oak Ridge National Laboratory
Pb-212: Lead-212
PET: Positron emission tomography
Ph. Eur.: European Pharmacopoeia
pmol: Pico moles
PSMA: Prostate Specific Membrane Antigen
QC: Quality control
Ra-223: Radium-223
Radio-TLC: Radio thin layer chromatography
RCP: Radiochemical purity
RCY: Radiochemical yield
ROI’s: Region of interests
SCK CEN: Belgian Nuclear Research Centre
SPECT: Single photon emission tomography
TAT: Targeted alpha therapy
Tb-149: Terbium-149
TFA: Trifluoroacetic acid
Th-227: Thorium-227
Tl-208: Thallium-208
TRIS: Tris(hydroxymethyl)methylamine
TRT: Targeted radionuclide therapy
U-233: Uranium-233
UV: Ultraviolet
